# Editorialets

**Published:** 1907-08

**Authors:** 


### Editorialets.

The Duplex "Talking Machine."—In these days of strenuous advertising so many things are boasted and boomed as the best that one has to be circumspect if he would not get caught. I got caught once on a talking machine. I have now a Duplex, and I am just tickled to death with it. It is the softest, clearest and most beautiful in tone of any of the many machines on the market. It can be made to play loud enough for the lawn or soft enough for the parlor. It combines all the excellencies of the best machines without any of the objections that attach to most of them, such as grating noise, etc. I confidently recommend it to the friends of the "Red Back" as the machine to buy. It is about half the price of machines controlled by the trust and is sent on seven days' trial. The Duplex is not in the combine. See ad, and write for illustrated book free.

A Four Thousand Dollar Practice for Sale.—Residence, barn, stables, buggy house, two wells, storm house, fruit garden, office and drug store, with a $500 stock of drugs, pair of matched buggy horses—blood bays, garden and three lots, all for $2500. Terms, cash, or part cash if desired. Address Dr. J. R., care of the Texas Medical Journal, Austin Texas.

An Embryo Substitutor.—A well known druggist in a Texas city took a country cub of 16 to learn the business. Seeing a lady go out without a purchase, he called the boy and asked what the lady called for. The boy said she wanted some H. V. C. "Why didn't you tell her we had some of our own make just as good, and some persons really preferred it?" asked the proprietor. Next day the lady asked for some hygienic toilet paper. "We are out of it," said the boy, "but we have something of our own make, just as good, for instance, we have fly paper, and—and—and—sand paper and some persons really prefer them."

To Manufacturers and Druggists.—A thorough druggist of large experience, registered, has traveled in Texas and Mexico, and speaks Spanish, wants to travel for a drug or proprietary house as detail man. Middle aged, a good canvasser and well acquainted with the medical profession. Endorsed and recommended by the editor of the "Red Back." Best of references; bond by the American Bond Company, if desired. Address the editor of this JOURNAL, Austin, Texas.

---

FOR PRICKLY HEAT or for mosquito bites, or for any kind of itching, I know nothing so good as Listerine, locally (diluted); and internally it is the best intestinal antiseptic known.—DR. DANIEL, Editor.

---

NO APPOINTMENTS YET.—At this date, August 1st, the Governor has made no appointments on the mixed board of medical examiners. The law is "effective from and after July 12th" (ult.), but there is no board. There are over two hundred applicants for appointment.

